# *Pandoraea pnomenusa* Superinfection in a Patient with SARS-CoV-2 Pneumonia: First Case in the Literature

**DOI:** 10.3390/idr14020025

**Published:** 2022-03-18

**Authors:** Diego Alejandro Cubides-Diaz, Natalia Muñoz Angulo, Daniel Augusto Martin Arsanios, Ana Lucia Ovalle Monroy, Daniel Ricardo Perdomo-Rodriguez, Maria Paula Del-Portillo

**Affiliations:** 1 Department of Internal Medicine, Universidad de La Sabana, Chía 140013, Colombia; danielmaar@unisabana.edu.co; 2 Faculty of Medicine, Universidad el Bosque, Bogotá 110121, Colombia; nmunoza@unbosque.edu.co; 3 Faculty of Medicine, Universidad de La Sabana, Chía 140013, Colombia; anaovmo@unisabana.edu.co (A.L.O.M.); danielpero@unisabana.edu.co (D.R.P.-R.); 4 Department of Internal Medicine, Clínica Universidad de La Sabana, Chía 250008, Colombia; maria.delportillo1@clinicaunisabana.edu.co

**Keywords:** *Pandoraea pnomenusa*, SARS-CoV-2, SARS-CoV-2 pneumonia, bacterial pneumonia, COVID-19, coinfection

## Abstract

*Pandoraea pnomenusa* is a Gram-negative bacterium of the *Pandoraea* genus and is mainly associated with the colonization of structurally abnormal airways. During the COVID-19 pandemic, many microorganisms have been associated with coinfection and superinfection in SARS-CoV-2 pneumonia, but so far, no coinfection or superinfection by *P. pnomenusa* has been reported. We present the first case describing this association in a previously healthy patient. Clinical manifestations, treatment, and outcomes are shown.

## 1. Introduction

The genus *Pandoraea* was first described in the year 2000, initially erroneously classified in the genera of *Burkholderia cepacia* and *Ralstonia paucula* or *picketti* [1]. This genus includes Gram-negative, aerobic, non-fermenting, non-spore-forming, and non-polar flagellated bacilli and is isolated from soil, water, and cystic fibrosis patients [2]. *Pandoraea pnomenusa* usually presents with resistance to multiple antimicrobials, including most beta-lactams. Among the resistance mechanisms, it presents an intrinsic OXA-type ß-lactamase and a *gyrB* gene [3]. Colonization of structurally abnormal airways is the usual presentation in humans. Though infection is rare, when it occurs, mortality rates are high, reaching up to 60% [2,4,5,6,7]. To our knowledge, this is among the first 10 cases of documented infection by this microorganism and the first one in a patient with SARS-CoV-2 pneumonia. 

## 2. Case Report

**Figure 1:**
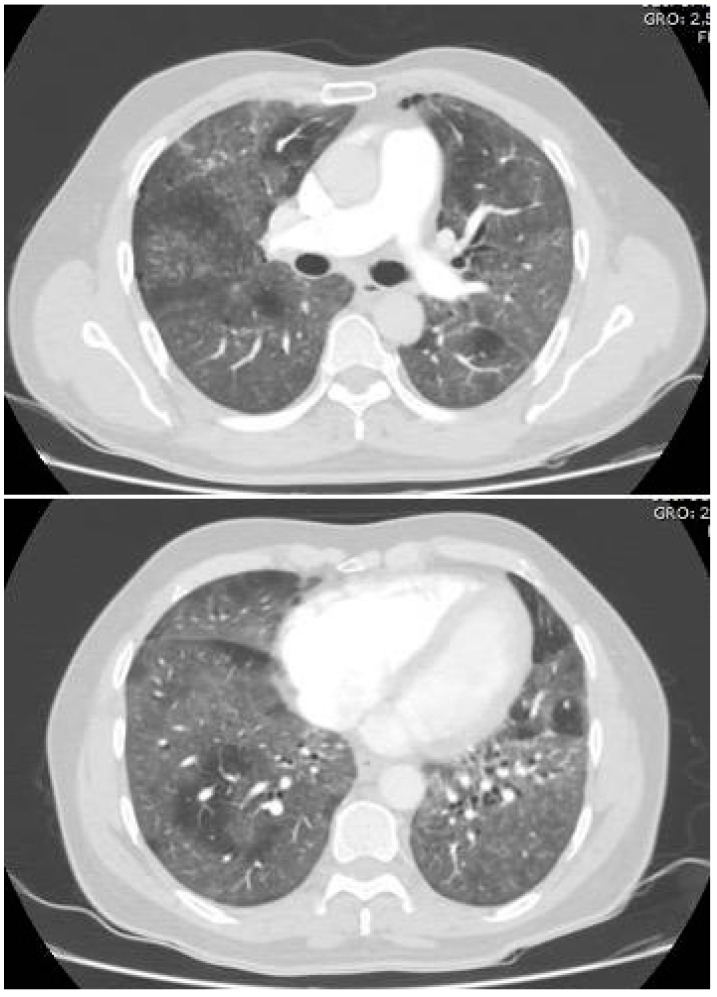
Chest computed angiotomography on admission showing diffuse ground-glass opacities with subpleural compromise and septal thickening. Negative for pulmonary embolism.

A 55-year-old male without medical records presented to the emergency department following a 10-day history of cough, shortness of breath, asthenia, and adynamia. Upon physical examination, lung auscultation revealed diminished respiratory sounds and crackles in both lungs, without other relevant findings. Initial laboratory findings are shown in Table 1, and admission chest angiotomography is shown in Figure 1. A SARS-CoV-2 antigen test was positive and bacterial coinfection was suspected, so corticosteroids with dexamethasone 6 mg daily and antibiotic therapy with ampicillin/sulbactam 3 g every 6 h were initiated. On day 3 of admission, the inflammatory response persisted, so he was admitted to the intensive care unit for clinical surveillance.

On day 5 of admission, he persisted tachypneic with labored breathing. Arterial blood pressure, temperature, and heart rate were normal, but high flow oxygen support was required to maintain an oxygen saturation of ≥90%. Later on, he worsened, requiring mechanical ventilation and vasopressor support. Cultures and respiratory molecular panels were collected. Respiratory nested multiplex polymerase chain reaction (PCR) was positive for *Klebsiella pneumonie* (BIN 10^5^) and *Serratia marcescens* (BIN 10^5^) without the detection of resistance genes. Antibiotic therapy was changed to cefepime at a dose of 6 g in a continuous infusion, with partial clinical response. Eight days after admission, the patient presented again with fever and a non-modulated inflammatory response; thus, antibiotic therapy was escalated to meropenem 6 g in continuous infusion plus linezolid 600 mg twice daily, under the suspicion of multidrug-resistant microorganisms. New cultures were taken, and *P. pnomenusa* was isolated from the respiratory secretion culture (35 × 10^5^ colony-forming units), identified by MALDI-TOF. Murray criteria were used, and contamination was ruled out since there was clinical deterioration and no other microorganisms were isolated.

**Figure 2:**
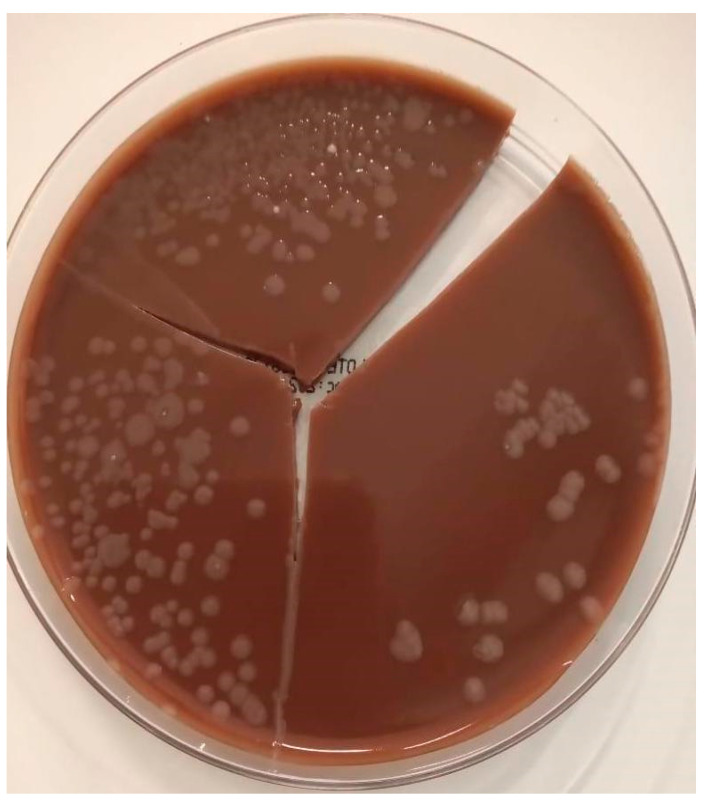
Chocolate agar with opaque white colonies of Gram-negative bacilli consistent with *P. pnomenusa*.

Since the mentioned microorganism does not have a breakpoint prespecified in CLSI (Clinical & Laboratory Standards Institute), disk diffusion susceptibility tests were run for ciprofloxacin and co-trimoxazole using a non-Enterobacterales breakpoint, and treatment was changed on day 10 to both agents at doses of 400 mg twice daily and 240/1200 mg thrice daily, respectively, with later adjustment of co-trimoxazole to renal function. The agar and resistance profiles are displayed in Figure 2 and Table 2. Three days after the initiation of the new antibiotic scheme, the inflammatory response started to wane and fever abated. On day 18 of hospitalization, tracheostomy was performed without complications; renal support was required with continuous venovenous hemodiafiltration due to persistence of acute kidney injury, volume overload, and anuria.

After the completion of 14 days of antibiotic therapy, the inflammatory response was completely eliminated. Eventually, respiratory weaning from mechanical ventilation was achieved, and renal replacement therapy was discontinued. He was discharged from the ICU on day 30 of hospitalization and continued respiratory and physical rehabilitation thereafter. Tracheostomy was decannulated on day 35, and the patient was discharged from the hospital on day 41 with supplementary oxygen and minimal sequelae, including physical and respiratory deconditioning and anxiety disorder related to the long hospital stay.

## 3. Discussion

*Pandoraea* is a bacterial genus described in the year 2000 by Coenye et al., who performed a taxonomic study on a group of bacteria identified as *Burkholderia cepacia*, *Ralstonia pickettii*, and *Ralstonia paucula*; these organisms were isolated from soil, water, and cystic fibrosis patients [1]. According to genotypic and phenotypic characteristics, they were classified in the new genus *Pandoraea* in order to group this series of specific strains, which were previously considered to belong to another genus with similar characteristics and phylogenetics. This group includes aerobic, non-fermenting, non-spore-forming, non-polar, flagellate Gram-negative bacilli [2]. Up to 10 species of *Pandoraea* have been described, but *P. apista*, *P. pnomenusa*, *P. pulmonicola*, and *P. sputorum* have been isolated most commonly from respiratory samples from cystic fibrosis patients and are related to death, poor clinical outcomes, and the potential to decrease lung function and increase pulmonary exacerbations [8,9]. *Pandoraea* spp. is also frequently associated with co-colonization by other respiratory pathogens, most notably *P. aeruginosa* [10]. 

To characterize this species, Robson et al. sequenced two *Pandoraea* strains isolated from patients with chronic respiratory diseases, both of which belonged to the *P. pnomenusa* species [10]. When compared to an environmental *P. pnomenusa*, these respiratory strains had 152 unique genes, most of which are associated with increased virulence and antimicrobial resistance [10]. Additionally, accurate genus and species identification by routine clinical microbiological methods is very difficult, and differentiation from *Burkholderia cepacia* complex organisms is problematic. Reliable identification requires 16S ribosomal DNA sequence analysis by PCR [1], which allows a significant reduction in the misidentification of *Pandoraea* spp. as *Burkholderia cepacia* complex isolates. Bayjanov et al. performed a genetic characterization of *Pandoraea* strains recovered from cystic fibrosis patients, revealing that some strains identified as *P. pnomenusa*, *P. sputorum*, *P. oxalativorans*, and *P. pulmonicola* belonged to another species of *Pandoraea*. Therefore, classification based solely on taxonomic characteristics of the *Pandoraea* genus could lead to misclassification of these microorganisms [9]. 

*P. pnomenusa* has been reported to be resistant to multiple antimicrobials, including most beta-lactams. Among the resistance mechanisms reported in some studies, an intrinsic OXA-type ß-lactamase, more precisely the *P. pnomenusa*-related OXA-62, and in a lower proportion, a *gyrB* gene, are the most important ones. OXA-62 is a class D carbapenem-hydrolyzing β-lactamase that appears to be specific in *P. pnomenusa,* allowing its easier identification. The preferred substrates of this enzyme are benzylpenicillin, amoxicillin, oxacillin, and the slow hydrolysis of carbapenems. It has no activity against ceftazidime, cefotaxime, or aztreonam [3]. 

Only five cases of infection by *P. pnomenusa* have been reported in the literature and are presented in Table 3. There are, however, more cases of colonization, mainly in patients with chronic pulmonary conditions such as cystic fibrosis. To our knowledge, this is the first case report of a respiratory superinfection in a patient without chronic pulmonary disease. The only predisposing factor was an acute SARS-CoV-2 infection, which represents a state of immunosuppression and hyperinflammation of the respiratory system. 

Regarding microbiological identification, initially, we used chocolate agar, which showed typical colonies (circular, grayish, and opaque), incubated aerobically at 37 °C for 48 h (two to three days are required for this microorganism to grow in a selective medium) [11]. Since phenotypic identification is not reliable, molecular techniques were applied using MALDI-TOF MS with the Microflex LT mass spectrometer with the FlexControl 3.0 and MALDI BioTyper 2.0 and 3.0 software programs, identifying the isolate as *P. pnomenusa* with values indicating secure genus identification and probable species identification. Subsequently, identification was performed with PCR and sequencing of 16S rRNA [12,13] using the primers: pnoF and pnoR with sequences (5′-3′) “CAGTGGGGAATTTTGGACAATGGGCGA” and “CGAGCACTCCCACCTCTCAGCAGGA”, respectively, specifically designed for the detection of *P. pnomenusa* with a product of 673 bp [1,14]. Antibiotic susceptibility testing was performed using antimicrobials that are presumably effective against this microorganism, namely, ciprofloxacin and co-trimoxazole. Since CLSI and EUCAST lack breakpoints for disk diffusion for non-Enterobacterales and lack specific breakpoints for *P. pnomenusa*, the decision to use these antimicrobials was extrapolated from the breakpoints for disk diffusion for *Burkholderia cepacia complex* and *Pseudomonas aeruginosa* using CLSI standards of 2020 [15]. Cases of *P. pnomenusa* infection reported in high-impact journals also extrapolated breakpoints from other non-Enterobacterales such as *P. aeruginosa* for determining sensitivity and making therapeutic decisions [5,7].

The mechanisms by which SARS-CoV-2 infection leads to airway immunosuppression are currently under investigation. It has been recognized that infected ciliated cells shed their ciliary axonemes, which disables mucociliary clearance, increases secretion accumulation, and probably promotes disease progression [16]. Additionally, the epithelium infection dampens the interferon response, especially alpha, beta, and lambda interferon gene transcription [16]. These changes are similar to those observed in patients with cystic fibrosis, where there is ciliary movement impairment leading to secretion accumulation and high salt concentration in the airways. This high salt concentration leads to the inhibition of antimicrobial peptides, including human β-defensins, and a subsequent increased infection susceptibility [17]. Additionally, two other mechanisms predispose to acquiring superinfections: hypermethylation of the interferon 1 gene, which leads to a decrease in interferon 1 concentrations in the airways [18], and the synthesis of pro-inflammatory cytokines, which are harmful to host cells [19]. This cytokine release syndrome, exhaustion of the immune system, and lung damage in patients with SARS-CoV-2 infection might lead to a state of immunosuppression, increasing susceptibility to superinfection by unusual microorganisms such as *P. pnomenusa*. These microorganisms, which are usually associated with colonization rather than infection in patients with structurally abnormal lungs, may gain clinical relevance in the course of coronavirus disease and could worsen the clinical course and outcomes in patients with acute respiratory infections. 

## 4. Conclusions

In the last 20 years, *P. pnomenusa* has been reported as the cause of microbial colonization of structurally abnormal airways. Infection is rare but has been associated with high mortality rates and usually requires antimicrobial therapy with non-beta-lactam broad-spectrum antibiotics. The association of these kinds of unusual infections with SARS-CoV-2 pneumonia is increasing; however, prior to this study, no superinfection with *P. pnomenusa* had been reported. More research is needed to elucidate the pathogenicity of bacterial superinfection in patients with COVID-19. 

## Figures and Tables

**Table 1:** Laboratory findings on admission and during hospitalization.

Laboratory	Laboratory Reference Ranges	Hospital Admission (Day 1)	ICU Admission (Day 3)	ICU Deterioration (Day 8)	Hospital Discharge (Day 41)
White blood cell count (×10^3^ uL)	5–10	9.09	9.79	12.6	11.99
Neutrophils (×10^3^ uL)	1.82–7.42	8.44 (92 %)	8.74 (89.4%)	11.7 (92.9%)	9.34 (77.9%)
Lymphocytes (×10^3^ uL)	1.5–4	0.40 (4.4%)	0.68 (6.9 %)	0.43 (3.2%)	1.42 (11.8%)
Hematocrit (%)	40–54	42	43	35.9	29
Hemoglobin (g/dL)	13.5–18	14.4	14	11.6	9.9
MCV (fL)	86–96	89.9	95.8	95.7	90.1
MCH (pg)	25–31	30.8	31	30.9	30.6
MCHC (g/dL)	32–38	34.3	32.3	32.3	33.9
RDW (%)	11–15	13.9	14.3	14.3	14.1
Platelets (×10^3^ uL)	150–450	257	337	280	356
C-reactive protein (mg/dL)	0.6–5	36.4	250	250.4	26
Blood urea nitrogen (mg/dL)	8–23	50.2	45.9	46.5	34.8
Creatinine (mg/dL)	0.67–1.17	1.11	1.54	1.54	1.52
D-dimer (ng/mL)	190–499	4610	6400	-	-
pH	7.39–7.47	7.48	7.23	7.34	7.5
PCO2 (mmHg)	29.4–39.8	29	58	50	41
PO2 (mmHg)	55.2–74	65	77	67	84
BE (mmol/L)	−3–3	−0.8	−3.3	0.6	8.1
HCO3 (mmol/L)	19.6–25.6	21.3	24.3	27	31.3
Lactate (mmol/L)	0.6–2.1	1.9	1.2	0.4	-

**Table 2:** Antibiogram and resistance profile of *P. pnomenusa* isolated from respiratory secretion.

Microorganism	Colony-Forming Unit (CFU)	
*Pandoraea pnomenusa*	35 × 10^5^	
**Antibiotic**	**Disk diffusion**	**Interpretation**
Ciprofloxacin (5 μg)	22 mm	Intermediate ^a^
Co-trimoxazole (1.25/23.75 μg)	30 mm	Sensitive ^b^

^a^ Pseudomonas aeruginosa CLSI breakpoint. ^b^ Burkholderia cepacia complex CLSI breakpoint.

**Table 3:** Cases of *P. pnomenusa* infection reported worldwide.

Author and Year	Title	Age and Gender	Clinical Records	Diagnosis	Bacteremia/Sepsis	Resistance Pattern	Management	Clinical Outcome
Falces—Romero I, et al. [4]—2016	Bacteriemia asociada a catéter por *Pandoraea* *pnomenusa* en un paciente pediátrico con leucemia aguda linfoblástica	10 months	Pre-B-cell acute lymphoblastic leukemia in a newborn, central venous catheter user.	Blood cultures, positive on day 4. MALDI-TOF identification	Yes/Yes	Sensitive: MIN, IPM	CMX + dexamethasone for 5 days, then CEF for 4 days, then IMI for 10 days	Recovery after 10 days of antibiotic
Ambrose M, et al. [5]—2016	*Pandoraea pnomenusa* Isolated from an Australian Patient with Cystic Fibrosis	26-year-old male	Cystic fibrosis with chronic infection by *P. aeruginosa,* managed with TOB, TZP, AZI	Sputum culture, positive on day 3. MALDI-TOF identification	No/No	Sensitive: IPM, CMX Resistant: CAZ, CIP, GEN, TOB, TZP, AMC, AZM, CRO, MEM, CL, TMP	Pre-hospital management with TOB + TZP + AZI. On day 4 of admission, management was changed to IMI + CMX, and one day later, VAN and CAS were added for a total of 5 days.	Death on day 11 of admission
Gawalkar A, et al. [6]—2020	Prosthetic aortic valve dehiscence following infective endocarditis by a rare bacterium–*Pandoraea pnomenusa*	42-year-old male	Mechanic valve replacement 20 years ago due to rheumatic valve disease	Blood cultures. Identification system and time to positivity not reported	Yes/Yes	Sensitive: LVX, MIN, CMX	VAN + TZP for 5 days, then MER + VAN + LVX for 9 days	Death on day 14 of admission
Stryjewski M, et al. [2]—2020	Sepsis, Multiple Organ Failure, and Death Due to *Pandoraea* *pnomenusa* Infection after Lung Transplantation	30-year-old male	End-stage pulmonary sarcoidosis complicated by nocardiosis and mycetomas, undergoing bilateral cadaveric lung transplantation. Pre-transplant management with prednisone 50 mg daily and itraconazole 100 mg twice daily.	Blood cultures on transplant day, positive at 48 h. Initially identified as *B. cepacia* but later correctly identified by PCR and RFLP	Yes/Yes	Sensitive: IPM Resistant: CAZ, CIP, TZP, CMX	Post-transplant management with CAZ + VAN + ABLC + GCV for 8 days, then CAZ was switched to MER and then to IMI	Death on day 17 of admission
Bodendoerfer E, et al. [7]—2021	Possible Prosthetic Valve Endocarditis by *Pandoraea* *pnomenusa* and Specific Virulence Mechanisms	37-year-old male	Intravenous drug user, native valve endocarditis with requirement for biologic prosthetic valve replacement. Received management with AMC and Isavuconazol	Peripheral blood cultures, positive at 23 h; PICC blood cultures, positive at 14 h. Incubation with BacT/ALERT Virtuo system and genomic sequencing with QIAseq FX DNA Library Kit system.	Yes/Yes	Sensitive: TZP, CEF, IPM, CIP, LVX, CMX Resistant: PIP, CAZ, CAZ-AVI, MEM, MV, AMK, GEN, TOB	TGC empirical, then TZP for 21 days, then CMX for 21 days	Recovery after 42 days of antibiotic. Control cultures were negative.
Cubides—Diaz D, et al.—2022	*Pandoraea pnomenusa* superinfection in a patient with SARS-CoV-2 pneumonia. First case in the literature.	55-year-old male	Acute severe SARS-CoV-2 infection with superinfection by *K. pneumonie and S. marcescens* treated with CEF.	Respiratory secretion culture, positive at 48 h. MALDI-TOF identification	No/Yes	Sensitive: CMX Intermedium: CIP	MEM + LZD empirical, then CIP + CMX for 14 days	Recovery after 14 days of antibiotic

Abbreviations: ABLC: amphotericin B lipid complex; AMC: amoxicillin + clavulanic acid; AMK: amikacin; AZI: azithromycin; AZM: aztreonam; CAS: caspofungin; CAZ: ceftazidime; CAZ-AVI: ceftazidime–avibactam; CEF: cefepime; CIP: ciprofloxacin; CL: colistin; CMX: co-trimoxazole; CRO: ceftriaxone; GCV: ganciclovir; GEN: gentamicin; IPM: imipenem; LVX: levofloxacin; LZD: linezolid; MALDI-TOF: matrix-assisted laser desorption/ionization–time of flight; MEM: meropenem; PCR: polymerase chain reaction; MIN: minocycline; MV: meropenem–vaborbactam; PICC: peripherally inserted central catheter; PIP: piperacillin; RFLP: restriction fragment length polymorphisms; TGC: tigecycline; TOB: tobramycin; TMP: trimethoprim; TZP: piperacillin–tazobactam; VAN: vancomycin.

## Data Availability

All relevant information is presented in the case report. Any additional data may be made available on reasonable request from the corresponding author.

## Abbreviations

CLSI	Clinical & Laboratory Standards Institute
COVID-19	Coronavirus disease 2019
EUCAST	European Committee on Antimicrobial Susceptibility Testing
MALDI-TOF	Matrix-assisted laser desorption/ionization–time of flight
PCR	Polymerase chain reaction
SARS-CoV-2	Severe acute respiratory syndrome coronavirus 2
